# Long-term response of metastatic hereditary leiomyomatosis and renal cell carcinoma syndrome associated renal cell carcinoma to bevacizumab plus erlotinib after temsirolimus and axitinib treatment failures

**DOI:** 10.1186/s12894-019-0484-2

**Published:** 2019-06-10

**Authors:** Inkeun Park, Young Sup Shim, Heounjeong Go, Bum Sik Hong, Jae Lyun Lee

**Affiliations:** 1 Division of Medical Oncology, Department of Internal Medicine, Gachon University Gil Medical Center, 1198 Guwol-dong, Namdong-gu, Incheon, 21565 Republic of Korea; 2 Department of Radiology, Gachon University Gil Medical Center, Incheon, Republic of Korea; 3 Department of Pathology, Ulsan University Asan Medical Center, Seoul, Republic of Korea; 4 Department of Urology, Ulsan University Asan Medical Center, Seoul, Republic of Korea; 5 Department of Oncology, Ulsan University Asan Medical Center, Seoul, Republic of Korea

**Keywords:** Hereditary leiomyomatosis and renal cell carcinoma, Metastasis, Bevacizumab, Erlotinib

## Abstract

**Background:**

Hereditary leiomyomatosis and renal cell carcinoma (HLRCC) is a rare hereditary kidney cancer syndrome in which affected individuals are at risk of skin and uterine leiomyomatosis and kidney cancer. HLRCC-associated kidney cancer is a lethal disease with a highly aggressive behavior, and there is no standard treatment option for metastatic disease.

**Case presentation:**

Here, we report a 29-year-old patient with a locally advanced HLRCC-assiciated RCC. He was administrated temsirolimus initially, then underwent surgical removal of kidney, retroperitoneal lymph nodes, inferior vena cava and tumor thrombi. Unfortunately, multiple liver metastases were confirmed 1 month after surgery, so axitinib was given but failed immediately. We tried bevacizumab plus erlotinib, which achieved long-term good response lasting more than 18 months. He is alive with disease and maintains bevacizumab plus erlotinib treatment.

**Conclusion:**

The promising results obtained in this patient suggest that combined bevacizumab plus erlotinib may offer a valid treatment option for advanced HLRCC-associated kidney cancer, even after failures of mTOR inhibitor and/or VEGFR TKI based therapies.

## Background

Hereditary leiomyomatosis and renal cell carcinoma (HLRCC) syndrome is a rare autosomal-dominant hereditary syndrome caused by germline mutation in the fumarate hydratase (*FH*) gene [1]. The phenotypic manifestations of this syndrome include cutaneous leiomyomatosis, uterine leiomyomatosis, and renal cell carcinoma. Renal cell carcinoma in HLRCC has a penetrance rate of 10–20%, and used to be categorized as a form of type 2 papillary RCC, but now is classified separately as HLRCC-associated RCC [2]. This type of RCC is a lethal disease with tendencies toward early metastasis and rapid progression, and thus, immediate surgical excision is recommended after its detection even when tumors are small [3]. Currently, the only curative option for HLRCC-associated RCC is early detection by routine screening in affected individual and immediate surgery. No standard systemic treatment option is available for metastatic disease, though most medical oncologists prescribe vascular endothelial growth factor receptor tyrosine kinase inhibitors (VEGFR TKIs) or mammalian target of rapamycin (mTOR) inhibitors. Here we report a case of metastatic HLRCC-associated RCC, which exhibited long-term response to bevacizumab plus erlotinib after failure of temsirolimus and of axitinib.

## Case presentation

**Fig. 1:**
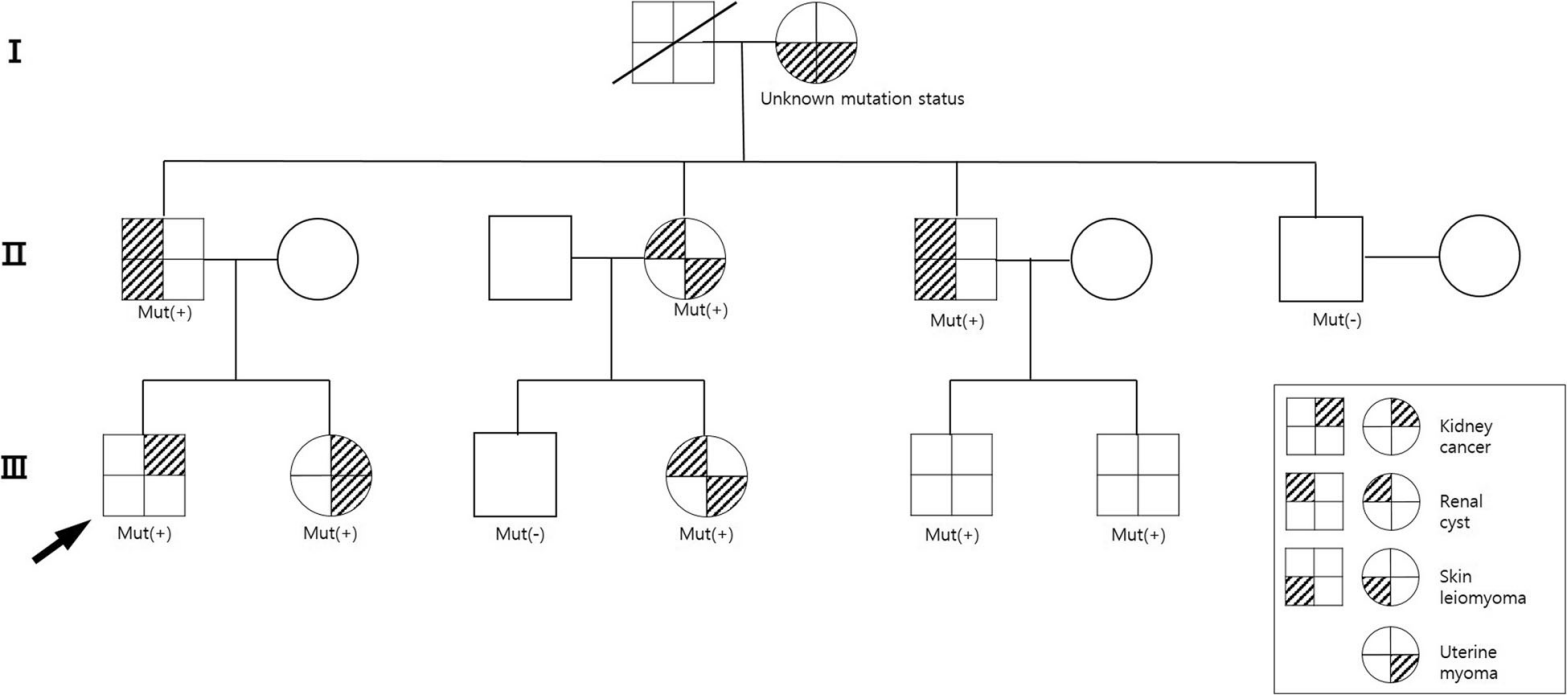
Patient pedigree showing individuals with uterine myoma, skin leiomyoma, renal cysts, and renal cell carcinoma

**Fig. 2:**
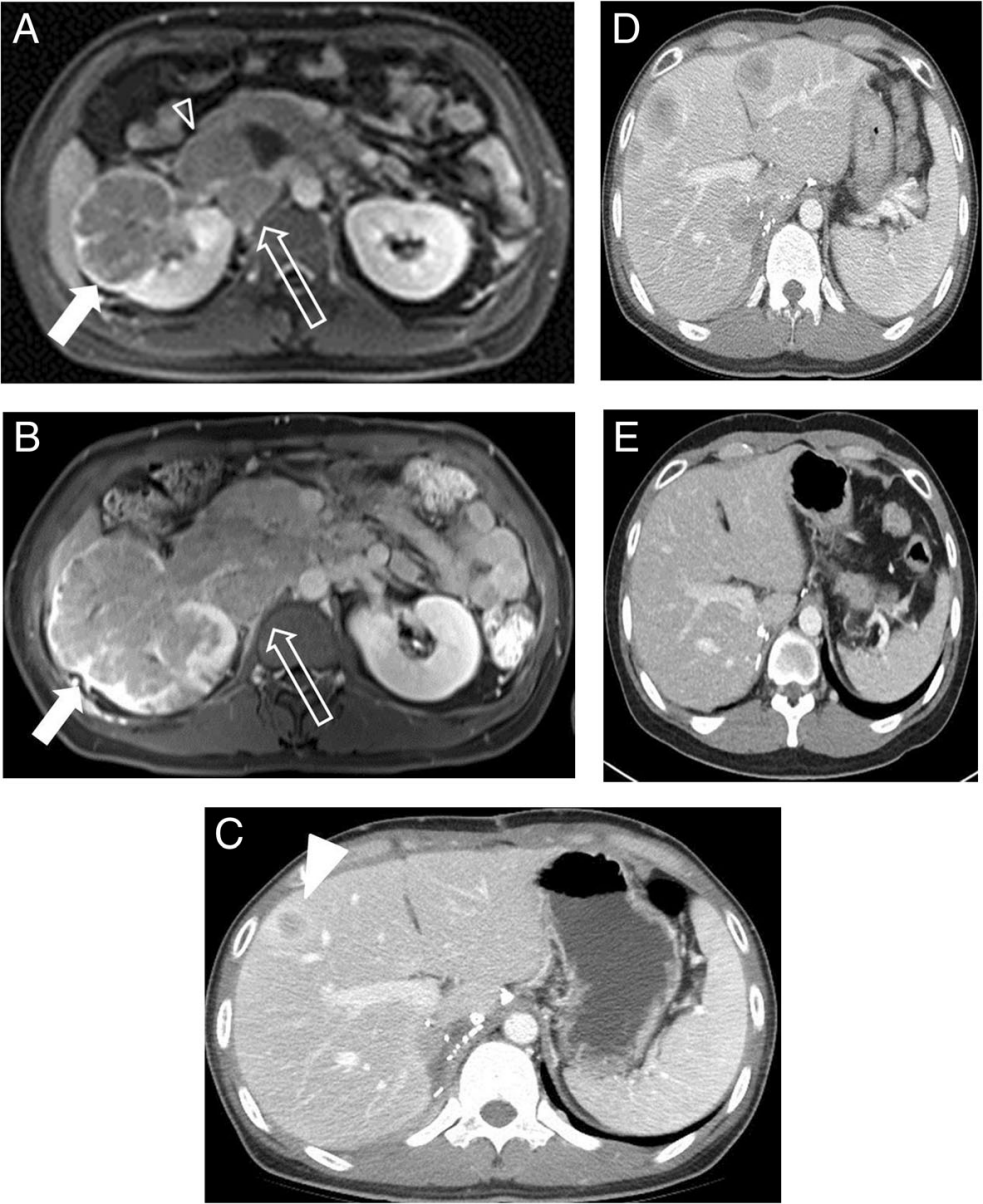
Serial CT and MRI images. **a** Axial fat saturated T1-weighted MRI image with contrast enhancement showing a lobulated contoured poorly enhancing tumor (arrow) in the right kidney. Tumor invasion was observed at IVC (arrow head) and metastatic lymph nodes (open arrow) were noted. **b** Despite temsirolimus treatment, primary tumor and metastatic lymph node size increased. **c** After radical nephrectomy, hepatic metastasis (arrowhead) was noted on a follow up CT scan, which (**d**) became aggravated despite axitinib therapy. **e** However, at 2 months after bevacizumab plus erlotinib therapy, the multiple hepatic metastases had nearly disappeared

A 29 year-old male presented a right flank mass and lower extremity edema in August 2015. He had no past medical history of illness, but a family history of uterine myoma in his sister, aunt, cousin, and grandmother and of skin leiomyomatosis in his grandmother, father, and uncle. His sister was later diagnosed with RCC in May 2016 (Fig. 1). Magnetic resonance imaging (MRI) of the abdomen revealed an 8 cm-sized mass in the right kidney that invaded the renal vein into infra-diaphragmatic IVC, obstructing and causing thrombosis of the infrarenal inferior vena cava and both iliac veins, with conglomerated lymph nodes (LNs) at retrocaval and aortocaval stations (Fig. 2a). Kidney mass biopsy revealed type 2 papillary renal cell carcinoma.

Because the massive thrombus and lymph nodes were deemed unresectable, he was administered temsirolimus from September 2015, but best response was stable disease. However, the primary mass and lymph node had enlarged in March 2016, indicating progressive disease (Fig. 2b).

**Fig. 3:**
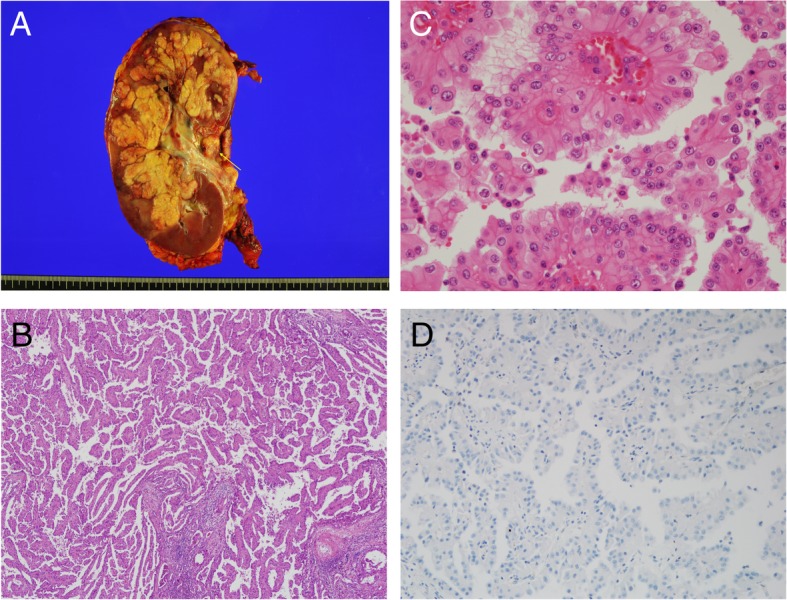
Gross and histologic characteristics of the primary tumor. **a** Nephrectomy specimen, the 11.8x7x7 cm sized well-demarcated, multilobulated mass in the right kidney had a whitish yellow, soft, friable papillary cut surface with renal vein thrombi (arrow). **b** Renal tumors exhibited infiltrative, tubulopapillary architectural growth intermixed with tumoral renal parenchyma (× 40). **c** Tumors were composed of large cells with abundant eosinophilic cytoplasm, large nuclei, and prominent inclusion-like eosinophilic nucleoli with perinuclear halos (× 400). **d** No expression of fumarate hydrate in tumor cells on immnohistochemical staining (× 200)

Because there is no standard treatment after temsirolimus in non-clear cell RCC and he maintained a good performance status, he underwent retroperitoneal lymph node dissection, IVC tumor thrombectomy, and radical nephrectomy in March 2016. Pathologic diagnosis was papillary type 2 RCC (Fig. 3). However, 1 month after surgery, follow-up CT demonstrated multiple liver metastases (Fig. 2c). Axitinib was started in May 2016, but the disease progressed, in liver, retroperitoneal LNs, and spine (Fig. 2d).

Considering his age at onset and family history of skin disease and uterine myoma, and tumor histology, HLRCC was suspected, and thus he and his family underwent germline *FH* mutation testing, which demonstrated the presence of mutation in *FH* exon 5 (c.688A > G, p.Lys230Glu). Although this specific mutation has not been reported in HLRCC, mutation in *FH* c. 689 A > G (p.Lys203Arg) had been reported to be pathogenic (rs752232718), and thus, we considered his kidney cancer was HLRCC-associated RCC. Immunohistochemical staining with anti-FH antibody (mousemonoclonal, clone J-13, 1:10000, SC-100743, SANTACRUZ, CA, USA) demonstrated no expression of FH in tumor cells (Fig. 3d). Based on a preliminary report, in which it was suggested bevacizumab and erlotinib in combination may be effective in HLRCC-associated RCC [4], we administrated bevacizumab (10 mg/kg every 2 weeks) and erlotinib (150 mg daily) from June 2016. After treatment, metastatic lesions in liver, LNs, and bone decreased rapidly, achieving partial response (Fig. 2e). As of Dec 2017, 18 months after start of bevacizumab plus erlotinib, this good response is maintained and the patient remains symptom free.

## Discussion and conclusions

In this case, we report long lasting response to bevacizumab plus erlotinib after temsirolimus and axitinib had both failed. Currently, temsirolimus is the only treatment option in non-clear cell RCC (nccRCC) that prolonged overall survival (OS) in a randomized controlled phase 3 trial [5]. However, this trial was not specifically designed for nccRCC, and included mostly clear cell RCC patients with poor prognostic risk group (*n* = 502, 80%), while the remaining 20% was composed of non-clear cell or indeterminated histology RCC (*n* = 124, 20%). Although statistically significant, the efficacy of temsirolimus was not impressive in this trial, as median OS was 10.9 months in the temsirolimus group compared to 7.3 months in the interferon group. Other than temsirolimus, VEGFR TKI can be an option. The randomized phase 2 trials, A Randomized Phase II study of Afinitor vs. Sutent in Patients With Metastatic Non-Clear Cell Renal Cell Carcinoma (ASPEN) and Everolimus Versus Sunitinib Prospective Evaluation in Metastatic Non-Clear Cell Renal Cell Carcinoma (ESPN), showed sunitinib appears to be more effective and numerically better than everolimus in nccRCC [6, 7]. Although sunitinib is the most widely studied VEGFR TKI in patient with nccRCC, the best VEGFR TKI for nccRCC have not been determined, as phase II trials on pazopanib and axitinib for nccRCC showed similar or better outcome compared to sunitinib [8, 9].

Trials on temsirolimus and VEGFR TKIs were conducted on a mixture of nccRCC variants, which included sarcomatoid, papillary type 1, papillary type 2, chromophobe, unclassified, and MiT family translocation RCC. Furthermore, multi-omics has recently revealed these variants are completely different disease entities with unique molecular pathobiologies [10, 11]. Therefore, ideally, a genomics-based clinical trial is required to determine the best treatment option for each of nccRCC variants. As HLRCC-associated RCC is a very rare, it is unlikely that any large-scale clinical trial dedicated to this cancer will ever be performed.

HLRCC is caused by a germline mutation in *FH* which encodes fumarate hydratase that converts fumarate into malate in the Krebs cycle. Consequently, HLRCC-associated RCC exhibits an impaired Krebs cycle and characteristic dependency on aerobic glycolysis. As fumarate accumulates, increased levels of fumarate inhibit hypoxia-inducible factor (HIF) prolyl hydroxylase which facilitates degradation of HIF-1α and HIF-2α. As a result, stabilization of HIF-1α leads to increased level of VEGF and GLUT1, which are necessary for aerobic glycolysis [12]. A mechanism-based clinical trial of bevacizumab plus erlotinib in papillary renal cell carcinoma is currently underway (NCT01130519). Interim results are promising, especially in patients with HLRCC-associated RCC [4]; response rate and median progression-free survival were 29% and 7.4 months, respectively, in non-hereditary papillary RCC, whereas 60% and 24.2 months, respectively, in HLRCC-associated RCC.

In conclusion, we suggest bevacizumab plus erlotinib be considered a treatment option in patients with metastatic HLRCC-associated RCC, even after failures of mTOR inhibitor and/or VEGFR TKI based therapies.

## Data Availability

Not applicable.

## Abbreviations

FH: Fumarate hydratase
GLUT: Glucose transporter
HIF: Hypoxia-inducble factor
HLRCC: Hereditrary leiomyomatosis and renal cell carcinoma
mTOR: mammalian target of rapamycin
RCC: Renal cell carcinoma
VEGFR TKI: Vascular endothelial growth factor receptor tyrosine kinase inhibitor
